# Osseodensification enables bone healing chambers with improved low-density bone site primary stability: an in vivo study

**DOI:** 10.1038/s41598-021-94886-y

**Published:** 2021-07-29

**Authors:** Rafael Coutinho Mello-Machado, Suelen Cristina Sartoretto, Jose Mauro Granjeiro, José de Albuquerque Calasans-Maia, Marcelo Jose Pinheiro Guedes de Uzeda, Carlos Fernando de Almeida Barros Mourão, Bruna Ghiraldini, Fabio Jose Barbosa Bezerra, Plinio Mendes Senna, Mônica Diuana Calasans-Maia

**Affiliations:** 1 Graduate Program, Dentistry School, Universidade Federal Fluminense, Niterói, Brazil; 2 Implantology Department, Universidade Iguaçu, Nova Iguaçu, RJ Brazil; 3 Oral Surgery Department, Universidade Iguaçu, Nova Iguaçu, RJ Brazil; 4 Post-Graduation Program in Dentistry, Universidade Veiga de Almeida, Rio de Janeiro, RJ Brazil; 5 Clinical Research Laboratory, Dentistry School, Universidade Federal Fluminense, Rua Mario Santos Braga, 28/4 Floor, Niterói, RJ Brazil; 6 National Institute of Metrology, Quality and Technology (INMETRO), Duque de Caxias, RJ Brazil; 7 Orthodontics Department, Dentistry School, Universidade Federal Fluminense, Niterói, RJ Brazil; 8 Dental Research Division, Dentistry School, Universidade Paulista, São Paulo, SP Brazil; 9 Laboratory of Bioassays and Cell Dynamics, IBB-UNESP, Botucatu, São Paulo, Brazil; 10 Implantology Department, UNIGRANRIO, Duque de Caxias, RJ Brazil

**Keywords:** Preclinical research, Implants

## Abstract

Primary implant stability is a prerequisite for successful implant osseointegration. The osseodensification technique (OD) is a non-subtractive drilling technique that preserves the bone tissue, increases osteotomy wall density, and improves the primary stability. This study aimed to investigate the hypothesis that OD, through a wider osteotomy, produces healing chambers (HCs) at the implant-bone interface without impacting low-density bone primary stability. Twenty implants (3.5 × 10 mm) with a nanohydroxyapatite (nHA) surface were inserted in the ilium of ten sheep. Implant beds were prepared as follows: (i) 2.7-mm-wide using subtractive conventional drilling (SCD) technique (n = 10); (ii) 3.8-mm-wide using an OD bur system (n = 10). The sheep were randomized to two groups, with samples collected at either 14-(n = 5) or 28-days (n = 5) post-surgery and processed for histological and histomorphometric evaluation of bone-implant contact (BIC) and bone area fraction occupancy (BAFO). No significant group differences were found with respect to final insertion torque and implant stability quotient (*p* > 0.050). BIC values were higher for SCD after 14 and 28 days (*p* < 0.050); however, BAFO values were similar (*p* > 0.050). It was possible to conclude that the OD technique allowed a wider implant bed preparation without prejudice on primary stability and bone remodeling.

## Introduction

Dental implants failure may be caused by local (low-density bone, compromised bone volume, and immediate implant placement) and systemic factors (systemic diseases, titanium allergy and tobacco use)^1–3^. Remodeling at the bone-implant interface requires the mechanical engagement of the dental implant with bone at the point of insertion, clinically defined as primary stability^4^. Several factors may influence this primary stability such as material biocompatibility, bone type and volume at the host site, loading conditions, surface technology (micro-nano topography and chemical composition), macrogeometry (implant body and thread design), and surgical preparation of the implant site^5^.

The use of biomimetic surfaces was observed in the early 1990s, particularly with hydroxyapatite (HA) coatings^6^, to improve the osteoconductive property of titanium for enhanced connection with the bone tissue^7,8^. However, a systematic review of clinical trials found similar long-term survival between HA-coated and uncoated titanium implants^9^. In fact, the use of plasma- sprayed HA coating may lead to implant failure caused by a rupture of the HA-titanium interface, leading to increased bacterial adhesion, and peri-implantitis^10^.

With the development of nanotechnology, biomimetic surfaces have migrated to the nanometric level. As a result, the emergence of nanohydroxyapatite (nHA) coatings allow for the use of HA to induce a chemical bond to the bone without the complications associated with plasma-sprayed^11^. Because bone tissue deposition on the surface of implant devices is strongly dependent on cellular interactions with the surface^12,13^, the nHA coating may accelerate osseointegration because it creates a hydrophilic surface with nanostructures-resembling the extracellular matrix of the bone tissue with respect to the size, shape, and crystallinity— which provides more substantial anchoring points at the surface for bone cells^14,15^.

The osseodensification (OD) concept was introduced to improve the primary stability of implants placed in low-density bone sites^16^. OD is a non-excavation osteotomy preparation method. In contrast to traditional standard drilling, OD compacts and auto-grafts bone in its plastic deformation phase^17^. It is a surgical instrumentation technique where the bone is compacted into open marrow spaces during drilling, increasing implant insertion torque through the preservation and densification of osteotomy site walls^16,18–23^. Because more bone particles will be present at the bone-implant interface when the implant bed is prepared, the use of OD maintains and conserves bone density, creates more bone-implant contact, and accelerates bone healing, consequently, enabling faster osseointegration^21,24^.

Due to the potential of biomimetic surfaces, creating a space between the implant surface and the bone tissue is recommended when drilling the implant bed as it facilitates the deposition of new bone at the interface^25^. This space, referred to as a healing chamber (HD),is accomplished by using a final drill with a diameter larger than the implant’s core diameter, but smaller diameter than the implant thread. Consequently, at the same time as the OD removes the necrotic bone layer created by the surgical instrumentation, the created space allows coagulum to accumulate at the interface, recruiting bone cells for faster bone formation^26,27^. However, the creation of an HC reduces the bone-to-implant contact (BIC), causing lower primary stability; hence, this procedure is generally not recommended for low-density bone.

The present study hypothesizes that the instrumentation when using the OD technique promotes a wider implant bed in low-density bone, enabling primary stability for implants with nHA coatings without impaired osseointegration. This study reports an in vivo biomechanical, histological, and histomorphometric analysis of nHA-coated dental implants.

## Results

This study was conducted on the ilium of sheep to evaluate two instrumentation techniques for nano-sized HA coatings dental implant installation.

**Figure 1:**
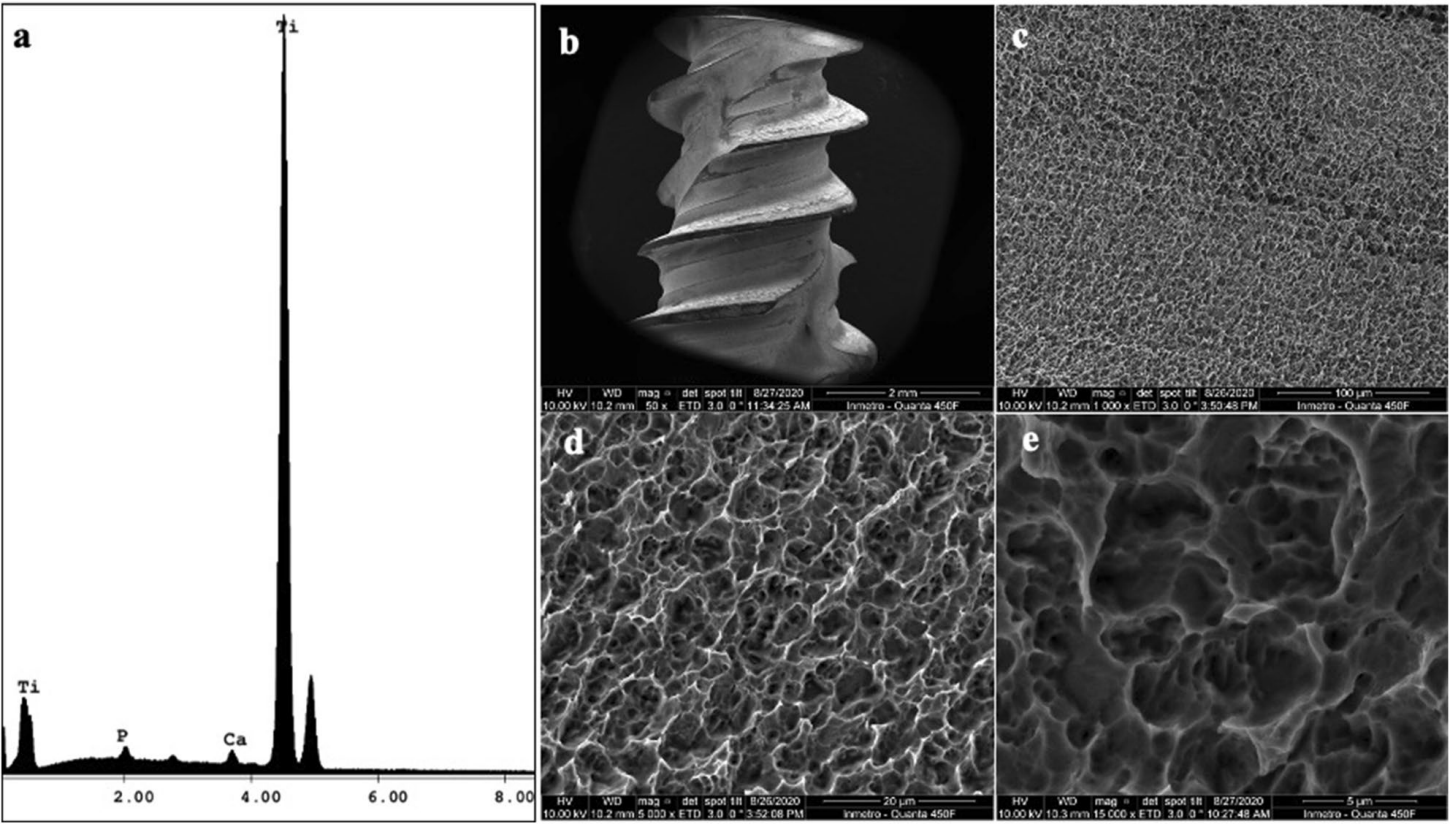
Scanning electron microscopy (SEM) micrographs of HAnano. (**a**) EDS results showing the peak of Ca and P; (**b**) implant geometry at 50 × magnification (scale bar = 2 mm); (**c**) implant surface at 1000 × magnification (scale bar = 100 µm); (**d**) implant surface at 5000 × magnification (scale bar = 20 µm); (**e**) implant surface at 15,000 × magnification (scale bar = 5 µm).

SEM micrographs of the implants surface showed a homogeneous topography of the implant surface at 50 ×, 1000 ×, 5000 ×, and 15,000 ×, and the energy-dispersive X-ray spectroscopy (EDS) showed the presence of calcium (Fig. 1).

In the present research, SCD and OD groups presented final IT values above 60 N-cm. The implant stability quotient was above 76, and the median for both groups was 75. (Table 1).

**Table 1:** Final insertion torque and implant stability quotient values for both surgical techniques: subtractive conventional (SCD) and the osseodensification (OD) drilling groups.

Procedure	Insertion torque (N/cm)*			Implant stability quotient (ISQ)*		
	Minimum	Median	Maximum	Minimum	Median	Maximum
SCD	60	70	80	69	75	76
OD	60	80	80	70	75	76

*Results represent average of five samples calculated for each group for IT and implant stability in minimum, median, and maximum.

**Figure 2:**
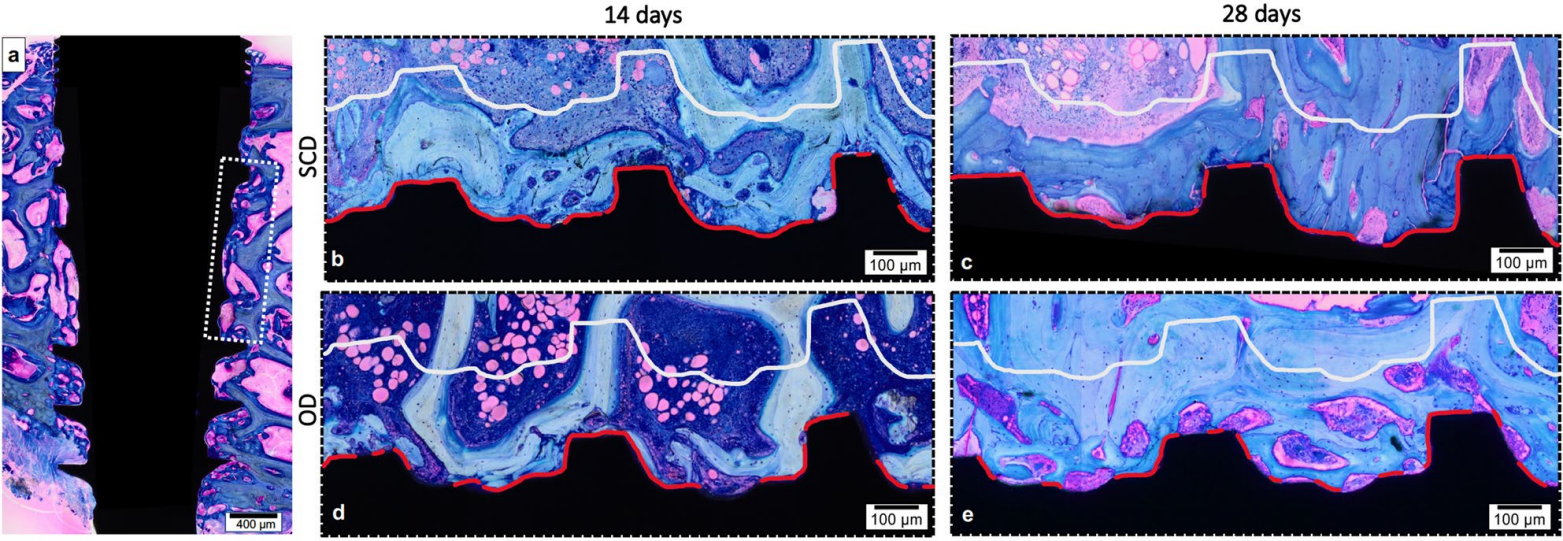
Illustration of histomorphometry methodology. (**a**) the area of interest for BAFO and BIC evaluation was determined from the first thread of the implant to the fourth thread’s beginning (dashed rectangle). The red line delimitation was used to determine the BIC value, which was later transformed into a percentage. The bone area fraction occupancy (BAFO) analysis was calculated after replication the design line of the implant profile 270 µm away from this profile. (**b**) SCD group after 14 days; (**c**) SCD group after 28 days; (**d**) OD group after 14 days, and (**e**) OD group after 28 days. Stain: Toluidine Blue and Acid Fuchsin stained. Scale bar: 100 μm.

The histological analyses of non-decalcified sections allowed the assessment of the biological response to the tested surgical techniques, the area of interest for BAFO and BIC evaluation was determined and drawn, from the first thread of the implant to the fourth thread’s beginning (Fig. 2a). Both groups indicated peri-implant bone regeneration. After 14 days of surgical procedure, the SCD group presented newly formed bone around the implants’ threads demonstrating an evident bone–implant contact (Fig. 2b). The OD group presented a similar reaction after 14 days, presenting new bone trabeculae islands surrounded by connective tissue permeating the implant surface (Fig. 2d). After 28 days, in both groups, newly formed bone around the implants was clearly apparent, and several areas of direct BIC were observed in a time-dependent fashion. The SCD presented extensive remodeling around the implant with a larger area and advanced degree of bone maturity (Fig. 2c), when compared to the previous period. The bone remodeling pattern in the OD group also presented more organized and compact bone tissue showing larger trabeculae of newly formed bone compared to the first period (Fig. 2e).

**Figure 3:**
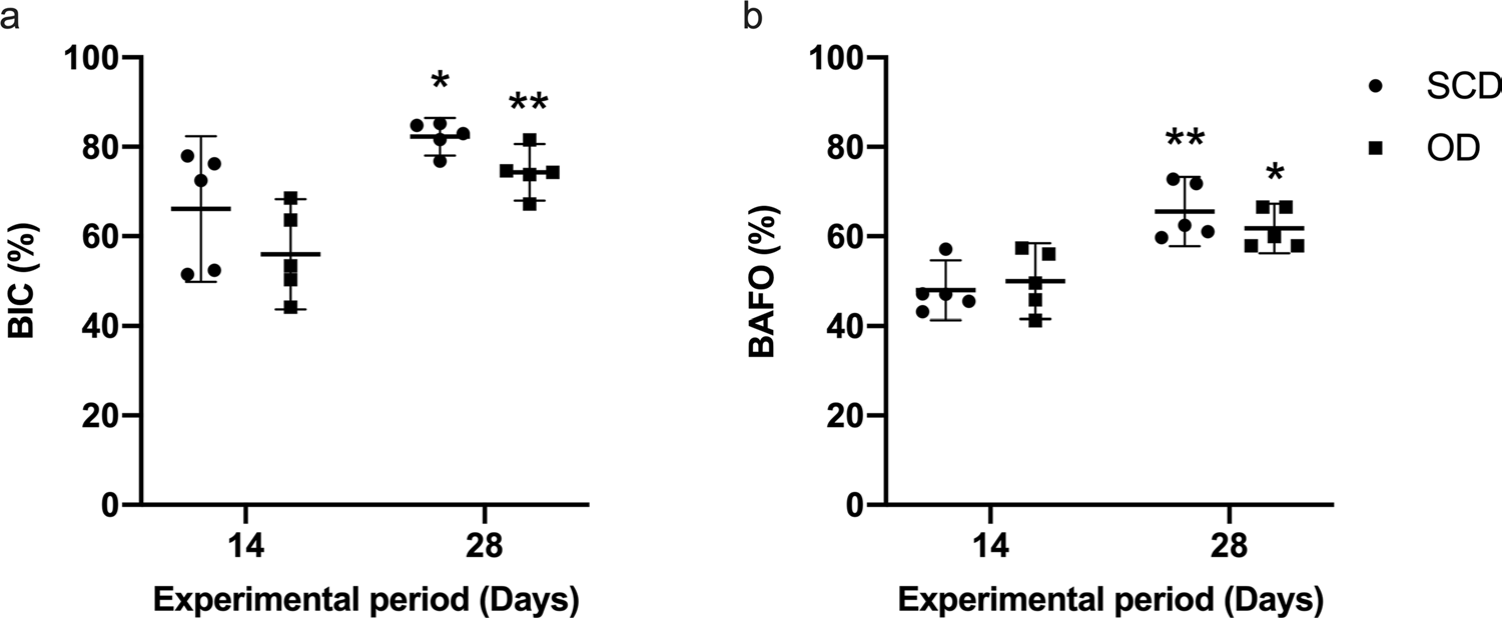
(**a**) The bone-to-implant contact (BIC) and (**b**) bone area fraction occupancy (BAFO) values of implants installed after subtractive conventional drilling (SCD) and osseodensification drilling (OD). * and ** indicates significant difference between the evaluation period (*p* < 0.05). The data are presented as mean and confidence interval (CI) at 95% of confidence.

A time-dependent increase of BIC and BAFO was observed in SCD (*p* = 0.002) and OD (*p* = 0.006) groups (Fig. 3); however, no significant between-group differences were identified (*p* > 0.05). After 14 days of healing, BIC values were 66.09% (CI: 49.80– 82.37) and 55.97% (CI: 43.63–68.31) for SCD and OD (Fig. 3a), respectively, whereas BAFO yielded 47.96% (CI: 41.29–54.64) and 49.99% (CI: 41.52–58.46) (Fig. 3b). After 28 days of healing, BIC values were 82.27% (CI: 78.08–86.47) and 74.30% (CI: 67.99–80.61) for SCD and OD (Fig. 3a), respectively, whereas BAFO yielded 65.53% (CI: 57.80–73.27) and 61.76% (CI: 56.24–67.29) (Fig. 3b).

**Figure 4:**
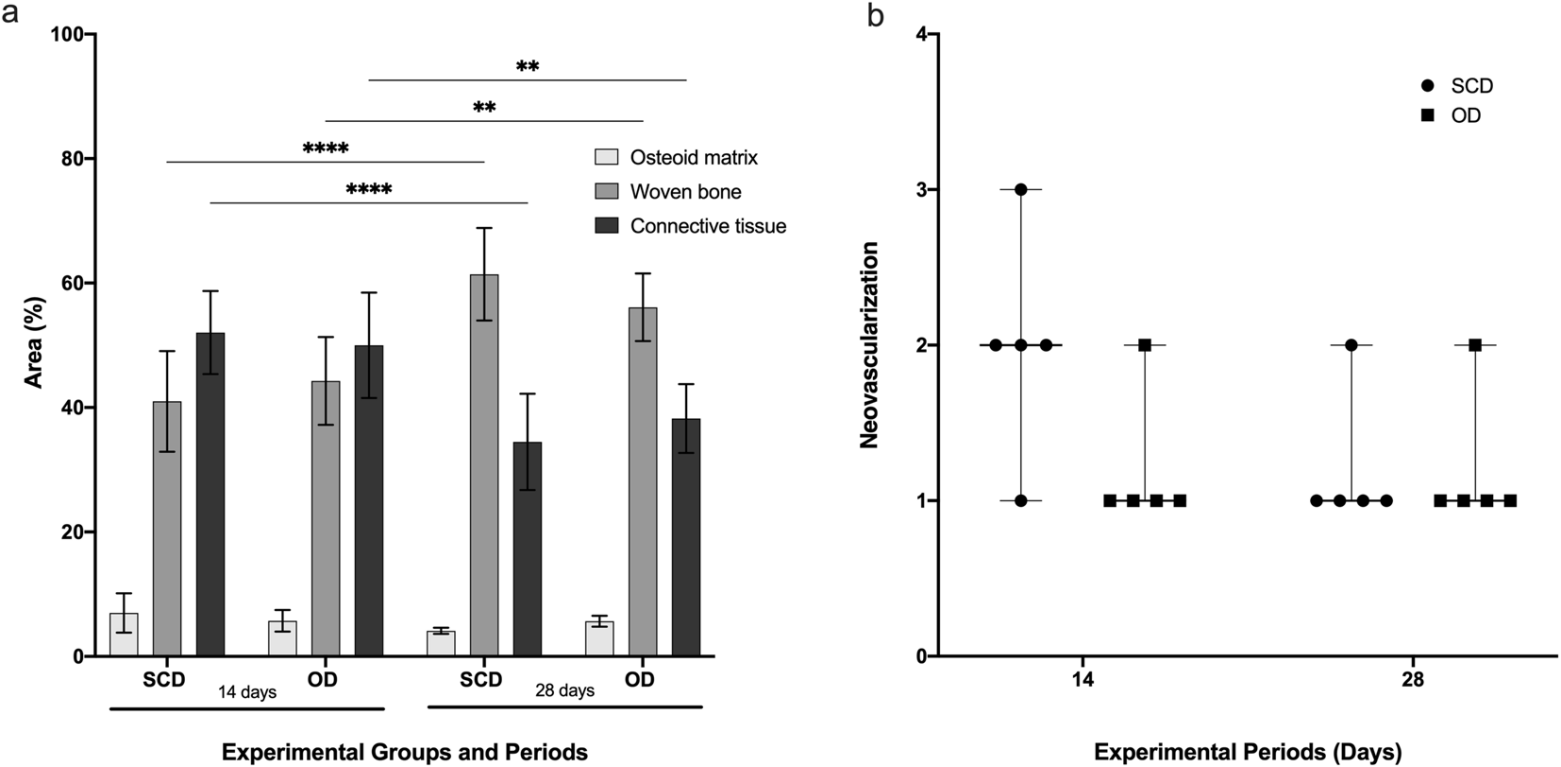
(**a**) Woven bone, osteoid and connective tissue percentage in the SCD and OD groups. The values are presented as mean ± confidence interval (n = 5). (**b**) The neovascularization was evaluated using the scores system from ISO 10993-6/2016. There was no difference between SCD and OD in the osteoid, wave bone, connective tissue percentage, and neovascularization (*p* > 0.05).

The results of the between-group comparison revealed no statistically significant difference with respect to the amount of osteoid, woven bone, and vessels (Fig. 4a,b).

After the Shapiro–Wilk test, the groups were submitted to statistical analysis of One-way ANOVA and Tukey post-test (*p* < 0.05) to evaluate the differences between groups and time points according to the presence of woven bone, osteoid and connective tissue percentage. The woven bone increased from 14 to 28 days while the volume of connective tissue decreased (*p* < 0.005). There was no difference between SCD and OD in the osteoid, wave bone and connective tissue percentage (*p* > 0.05).

The neovascularization was evaluated using the scores system from ISO 10993-6/2016: no capillary proliferation (0); minimal capillary proliferation, focal 1–3 buds (1); groups of 4–7 capillaries with supporting fibroblastic structure (2); broad band of capillaries with supporting structures (3); extensive band of capillaries with supporting fibroblastic structures (4). The values are presented as median ± confidence interval (n = 5). After Mann–Whitney test, no statistical difference was observed between groups and periods after 14 and 28 days (*p* > 0.05).

**Figure 5:**
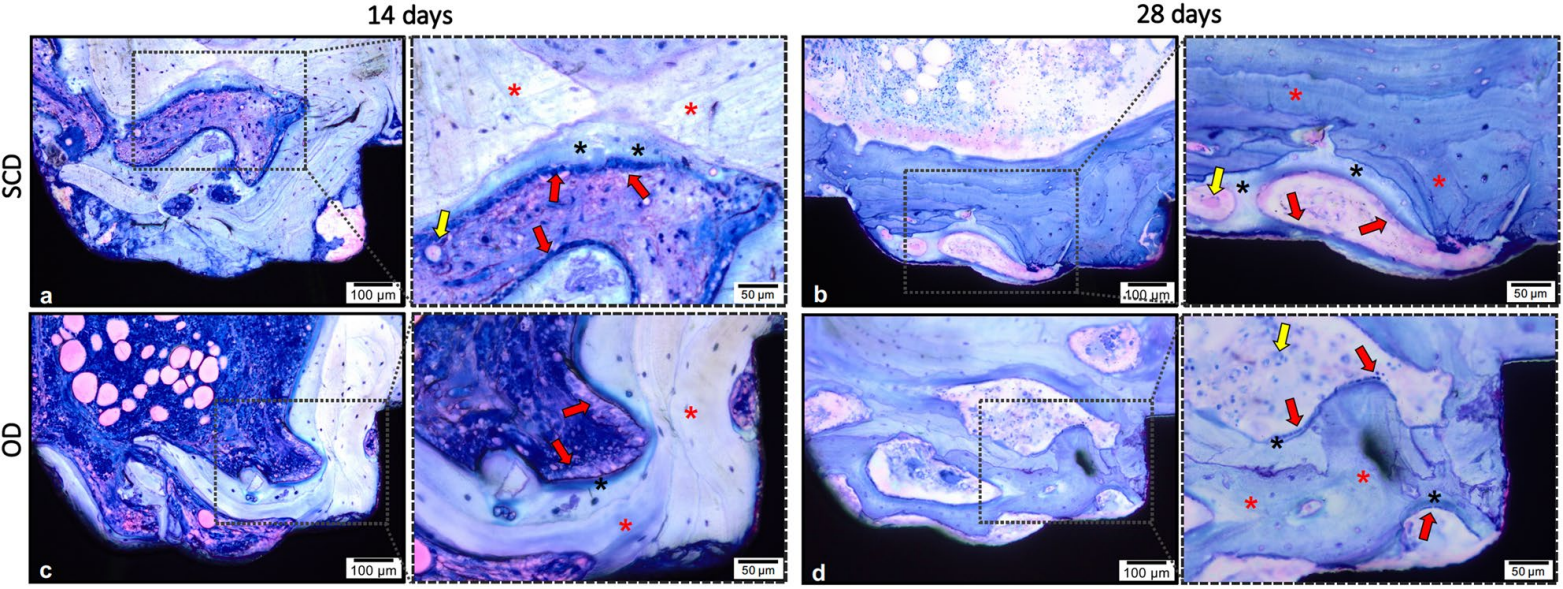
Representative photomicrographs of wound healing to different surgical drill techniques. The dashed area is the magnification of the square, which allows the visualization of the vessels (yellow arrow), woven bone (red asterisk), osteoblasts paving (red arrow) and below, the osteoid matrix (black asterisk). SCD (**a**, **b**) and OD (**c**, **d**) 14 and 28 days after implantation, respectively. The area corresponds to the third most coronal thread. Stain: Toluidine Blue and Acid Fuchsin. Magnification: 20 × and 40 × ; Scale bar: 100 µm and 50 µm.

Figure 5 shows the presence of osteoid matrix limited by osteoblasts; the different colors of osteoid matrix (light blue) and osteoblast (blue navy) allowed the quantification of osteoid matrix in both groups, which showed no differences between the groups.

**Figure 6:**
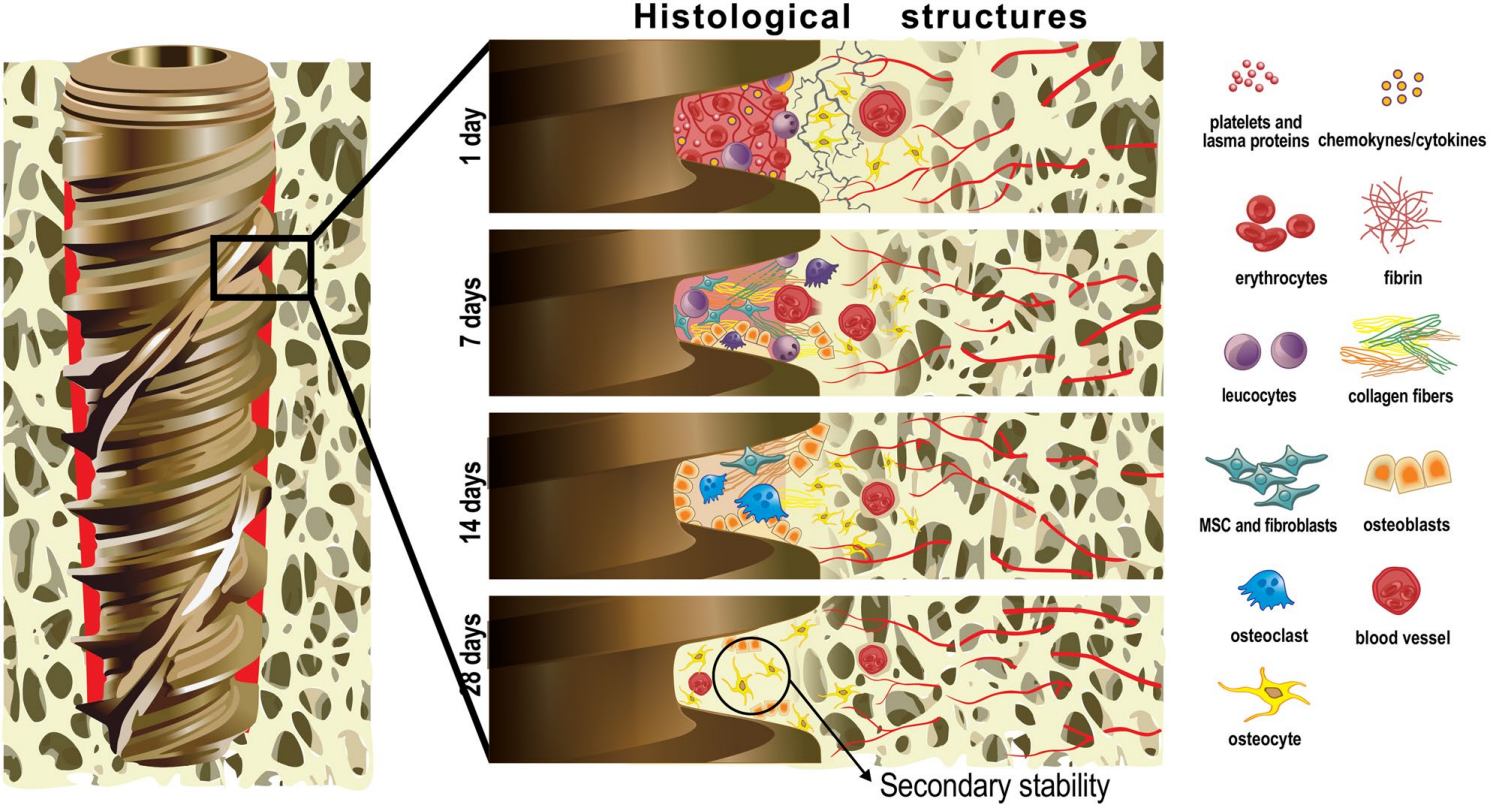
Graphical representation of timeline of osseointegration of dental implants concerning cellular events from day 1 to 28 after implantation. It is possible to see the presence of a well-defined bone-implant interface after 28 days of implantation. The cellular information of days 1^28^ and 7^29^ was based on previous studies. The authors acknowledge Dr. Helder Valiense for the help in making the schematic figure.

The histological events at the HC are summarized in Fig. 6. The cascade of cellular events that occur between the biological environment and the implant surface initially involves the presence of blood clotting with a thin layer of serum protein, which progresses to granulation tissue, followed by immature woven bone. The bone formation begins early, during the first week, through the promotion of osteoblast differentiation and the production of osteogenic factors, cytokines, and growth factors. The primary bone that includes trabecular of woven bone is substituted by parallel-fibered and/or lamellar bone and marrow. Between weeks 1 and 2, the bone tissue responsible for primary mechanical stability of the implant, immediately lateral to the implant region, is resorbed, and substituted by newly formed bone. After 4 weeks, secondary stability is established with a substantial number of osteocytes, as illustrated in Fig. 6.

## Discussion

The focus of the current study was to evaluate the primary stability and osseointegration of an nHA coated implant inserted using OD with a wider implant bed; the use of SCD was chosen as control. The sheep ilium model characterized by low-density bone was selected for its use in this study. The results revealed no between-group differences with respect to BIC and BAFO values, confirming the null hypothesis of the study.

Whereas bone healing in many animal species is recognized to be faster than in humans, sheep are considered to have a bone healing rate similar to humans^30^ and have been previously established as useful models for human bone turnover and remodeling activity^31^. Sheep present advantages, compared with other experimental models, for evaluating orthopedic and dental implant systems, such as acceptance by the animal research society and easy management^32,33^, similarity to humans with respect to body weight^34^, composition, metabolism, and bone remodeling and regeneration time^32,35^. Furthermore they have bones with dimensions suitable for the deployment of implant systems and devices of bone fixation that are designed for humans^32^, and enable evaluation of up to 12 implants (in the final size for marketing) per animal^34^. Early osseointegration is still considered a challenge in areas with the most trabeculated bone (bone type IV). The sheep ilium was used as the animal model because this region is considered to be a low-density bone^21,36,37^, it had already been used in other histomorphometric studies involving dental implants, an absence of postoperative morbidity^36^ and no need to euthanize the animals^38^. Once the bone quality was characterized (type IV), the anatomical region in the jaws was not considered because the presence of low-quality bone may be related to the individual physiology of each patient and not to the anatomical position in the jaws.

All previous animal studies aimed to investigate the bone–implant contact and bone area fraction occupancy at different time points and macro/micro geometries. Furthermore, two clinical trials^4,39^ aimed to compare the insertion torque and implant stability of implants placed via OD and SCD. The present study evaluated for the first-time histological events around nanohydroxyapatite dental implant coating using different drilling techniques.

The clinical approach for the installation of dental implants in low-density bone, such as the posterior region of the maxilla, typically consists of an underprepared implantation site to improve the implant’s primary stability so that osseointegration can proceed. On the other hand, implants presenting biomimetic surfaces, which can enhance the bone healing process, may benefited when HCs are present at the surface^40^. Furthermore, when the osteotomy is performed to allow the presence of the HCs at the bone–implant interface, it reduces the BIC. However, there is a gap of evidence concerning the ability of the biomimetic surfaces to overcome the inadequate primary stability induced by the production of the HCs. Hence, it is worth exploring whether a hydrophilic surface with nanostructures and associated with a wider osteotomy technique, producing HCs at the implant–bone interface, would produce sufficient bone to provide satisfactory initial implant stability.

Therefore, the present study was designed to verify whether the OD technique could be a viable approach for standardization of the HC in low-density bone when using dental implants with a biomimetic surface. The implants used in this study have a bioactive surface with nanostructured hydroxyapatite and the same macro-geometry, diameter, and length; therefore, the only difference between the groups was the instrumentation technique. In the OD group, the final implant bed was 3.3 mm, whereas in the SCD group, it was 2.7 mm. The wider implant bed in the OD group allowed for the creation of an HC.

The first prerequisite for osseointegration is adequate final insertion torque and the implant’s primary stability^13,41^. Both surgical techniques in the present study demonstrated adequate primary stability in the low-density bone, reflecting the special design of the implant to boost the primary stability^42^. Comparing the surgical techniques, similar IT and ISQ were identified for both groups, demonstrating that the densification of the interface promoted by OD compensates the use of an implant–implant bed discrepancy of 0.6 mm. The improvement of primary stability when using OD was also observed in previous studies^16,21,26,37,43–46^, regardless of the implant’s design or the implant surface^20,22,47^. Nevertheless, previous studies demonstrated that the densification of the bone interface does not guarantee adequate primary stability ex-vivo^18^ and in vivo^48^ because of high interfacial stresses that caused fractures and triggered a prolonged period of bone resorption. However, it was the first time that such a small implant–implant bed difference had been evaluated, and no fibrous tissue formation was identified at the bone–implant interface.

As expected for a wider implantation bed, BIC results at 14 days indicated no statistical differences. In contrast, Pantani et al. demonstrated in vivo study in dogs that milling with a final diameter 0.2 mm narrower than the implant produces a bone–implant contact similar to subtractive conventional osteotomy, with a final diameter 0.8 mm smaller than the implant^40^. When SCD is compared to OD at same final diameter, previous studies, using the same animal model, have shown that this technique improved the bone volume around dental implants^19,49,50^. In contrast, other studies did not find any healing impairment related to the instrumentation^47^. Unfortunately, in the current study design, the BIC was not assessed at the time of implant insertion, which would have allowed for assessment of the histological variation in 0 to 14 days of healing.

Following histomorphometric analysis of BAFO, the implant–profile design was then duplicated and aligned at 270 µm in the long axis of implant, thus completing the total area of interest. This methodology was based on a previous study that used 200 µm; in our study, 270 µm was used to enlarge the area of interest^51,52^. When BAFO is observed, it is possible to note that a faster bone healing in the OD group again compensates for the larger osteotomy. The bone interface was furthest from the implant surface in the OD group at the moment of implant insertion, and after 14 days of bone healing, BAFO values were similar to the SCD group. In contrast, previous data reported similar bone healing when using OD drills in subtractive (clockwise rotation) and densification (counterclockwise rotation) modes^47,49^. Because a 3.8 mm-wide implantation bed without OD group was not evaluated, it was not possible to conclude whether the faster healing would be a consequence of the HCs at the interface, as proposed by other authors^24,49^, or because there was a denser bone–implant interface.

After 28 days of healing, BIC and BAFO values were similar in both SCD and OD groups. After the same period, a higher between-groups difference was reported in a previous study using a pig model, in which BIC results were obtained for implants inserted in beds prepared with OD (62.5%) and compared to implants inserted in beds prepared by the osteotome technique (31.4%) in the mandibular crest^45^. However, the initial interlocking due to the implant geometry was higher in the present study, which can overlap the benefit of OD in improving bone density at the interface. After 28 days, it was possible to observe the secondary bone and connective tissue around the implants; hence, the longest experimental period in this study was 28 days.

According to Trisi et al.^53^, immediate implant loading can be recommended when IT is at least 45 N-cm, and ISQ is at least 68^53,54^. Here the median for IT was higher than 70 N-cm and the ISQ was 75 for both groups, allowing for both instrumentation techniques for the immediate implant loading.

The OD concept can be applied in clinical practice in situations such as promoting bone ridge expansion with enhanced primary stability and higher insertion torque values, minimizing implant dehiscence, fenestrations, and can be used for crestal sinus lift in a simple, safe, and predictable way with reduced morbidity^55^. Previous clinical studies have demonstrated improved of osseointegration^56^ and higher IT and RFA^17,55,56^. The present pre-clinical study adds new evidence through a histomorphometric analysis of the bone-implant interface occurring after 14 and 28 days of healing, quantifying the osteoid, woven bone, connective tissue, and vascular neoformation. Altogether, the results improve understanding of the osseointegration process through two different instrumentation techniques on a bioactive nano-hydroxyapatite surface.

Despite this study finding that a wider implant bed using OD is a viable approach in low-density bone, it is important to highlight that only one implant geometry was used. Therefore, the extrapolation of the results of the present study to other implant systems should be done with care. Although the ability of the biomimetic nHA implant surface together with the HC allows for adequate primary stability, extrapolation of the current results to other implant surfaces should be done with caution and long-term analyses are required to better understand the effect of OD with another implant surface on implant survival. Long-term analyses for assessing bone saucerization as a function of the osteotomy technique are strongly recommended. Within the limitations of this study, it can be concluded that OD technique performed with a wider surgical bed provided comparable levels of initial implant stability, BIC, and BAFO to the conventional subtractive under-drilling procedure without impairing the osseointegration.

## Methods

### Dental implants and scanning electron microscopy

This study used 20 titanium dental implants (3.5 mm in diameter and 10 mm in length) with a nano-sized crystalline nHA coating (Epikut Plus, S.I.N. Implant System, Sao Paulo, SP, Brazil). High-resolution scanning electron microscopy (SEM) images obtained with an FEI-Quanta 450 (Thermo Fisher Scientific, Waltham, MA, USA) revealed the surface topography of the implants, at an accelerating voltage of 10 kV; focal width of 3.0; and magnifications of 50 ×, 1000 ×, 5000 ×, and 15,000 ×. Energy-dispersive X-ray spectroscopy analysis determined each surface’s chemical composition at an acceleration voltage of 20 kV and focal width of 40 using an EDAX detector equipped with a dual-beam electron microscope (AMETEK Materials Analysis Division, Mahwah, NJ, USA) and the Genesis software program (EDAX, LLC, Mahwah, NJ, USA).

### Animal model

This in vivo study was approved by the Institutional Animal Care and Use Committee from Federal Fluminense University (protocol # 9531061119) following the Animal Research: Reporting of In Vivo Experiments (ARRIVE) and Planning Research and Experimental Procedures on Animals: Recommendations for Excellence (PREPARE) guidelines^57,58^. The animals were kept and operated at the Federal Fluminense University Farm School and were accompanied by a veterinarian with more than 20 years of experience. All experiments were performed between March and July of 2020.

The sample size was calculated using a *priori* power analysis based on the results from a previous study, which evaluated BIC in the same experimental animal model, to estimate the effect size^36^. Considering a type 1 error of 0.05 and power of 0.95, the two-tailed t-test determined a sample size of 5 implants per group/time point. In compliance with the reduction, refinement and replacement program^59^, the animals were also used for another study^36^. None of the animals were euthanized after the end of the present study.

Ten adult female Santa Ines sheep aged 2–4 years, with an average body weight of 37.05 kg (range 31–42 kg), were randomly allocated using the coin-toss method into two experimental periods (14 or 28 days of healing).

Each animal received two implants (one for each group) in the ilium, a low-density bone, with a wide bone area that simultaneously allowed the installation of multiple implants. The bone blocks can be collected without any morbidity for the animals in terms of locomotion and health. The implant position was randomly defined using the sealed envelope method, a surgical map of implant positioning inserted in the selected animal, ensuring a similar distribution into bone tissue for both groups. The inter-implant distance was at least 5 mm.

Before beginning the study, all animals presented good general health and physical condition after clinical examination by an experienced veterinarian. In the preoperative period, the animals received food composed of the pastures and, during the postoperative period, in addition to the aforementioned pastures, nutritional supplementation appropriate for sheep. Salt mineral water ad libitum was available during the entire experimental period. The animals were transferred from the field to the research center two weeks before the surgeries to avoid stress. The animals fasted for eight hours before the surgery.

### Surgical procedure and implants installation

The animals were given 0.05 mg/kg of acepromazine intravenously (Acepran; Vetnil, Louveira, Sao Paulo, SP, Brazil) and 0.2 mg/kg of diazepam intravenously (Diazepan; Teuto, Anapolis, GO, Brazil), as well as 0.4 mg/kg of morphine intramuscularly (Dimorf; Cristalia, Itapira, Sao Paulo, SP, Brazil) for premedication. After orotracheal intubation and ventilation, 4 mg/kg of propofol intravenously (Propofol Baxter; Baxter Hospitalar LTDA; São Paulo, SP, Brazil) was provided and sustained using 1% isoflurane (Cristalia, Itapira, SP, Brazil). Meanwhile, 4 mg/kg of lidocaine (Xylestesin; Cristalia, Itapira, SP, Brazil) and 0.1 mg/kg of morphine (Dimorf; Cristalia, Itapira, SP, Brazil) were used for epidural. block. The edges of the iliac crests were exposed through a horizontal skin incision of 5 cm in length. The skin and fascial layers were opened separately using a scalpel handle no. 3 (Bard Parker; Aspen Surgical, Caledonia, MI, USA) and blade no. 15 (Solidor; Lamedid, Osasco, Sao Paulo, SP, Brazil).

Two different instrumentation techniques for the preparation of the implantation bed were used: control group, subtractive conventional drilling (SCD) according to the implant manufacturer instructions for low-density bone (lance bur, 2.0 and 2.7 mm diameter tapered burs); and experimental group, OD drilling using multi-fluted tapered burs (2.0 mm pilot, 2.5, 3.0, and 3.3 conical burs) (Densah Bur; Versah, Jackson, MI, USA), with a final diameter larger than the implant core diameter. Drilling was performed with clockwise rotation for SCD and counterclockwise rotation for OD group at 1200 rpm under saline irrigation for both groups. Drilling for both groups was performed by the same operator (M.D.C-M.).

All implants were installed with the aid of a handpiece coupled to a drilling unit (BLM 600 plus; K Driller, Sao Paulo, SP, Brazil) under a profuse 0.9% sodium chloride solution (Sterile Saline Solution; Eurofarma, Rio de Janeiro, RJ, Brazil) and in low rotation (24 rpm) to avoid tissue necrosis due to overheating. The final insertion torque (IT) was recorded for each implant by the drilling unit. When the IT value was higher than 50 N-cm, an analogic wrench was used (S.I.N. Implant System, Sao Paulo, Brazil). The minimum, median, and maximum values for IT of the five samples were calculated for each group. The implant stability quotient (ISQ) was determined with an Osstell IDx device (Ostell/Integration Diagnostics; Gothenburg, Västra Götaland, Sweden), simulating mesiodistal and buccolingual measurements, and the average was recorded^60^. The minimum, median, and maximum values for ISQ of the five samples were calculated for each group.

After surgical procedures, all animals received 4 mg/kg of the analgesic Tramal (Tramadol; Pfizer, New York, NY, USA) and 0.5 mg/kg of the anti-inflammatory meloxicam (Meloxivet; Duprat, Rio de Janeiro, RJ, Brazil) over five days. Antibiotic therapy by intramuscular injection of 0.1 mL/kg of oxytetracycline (Terramicina; Pfizer, New York, NY, USA) was also used every 24 h for three days, including the day of the surgery. Oxytetracycline spray with hydrocortisone (Terra-Cortril Spray; Zoetis, Sao Paulo, SP, Brazil), and zinc oxide ointment with cresylic acid (Unguento Chemitec; Chemitec, Sao Paulo, SP, Brazil) together with silver spray (Aerocid Total; Agener União, Araçoiaba da Serra, SP, Brazil) was applied daily at the wound site to support healing and prevent local infection.

### Histological procedures

The sheep were submitted to the same anesthetic procedures following 14 and 28 days of healing. The bone blocks were collected with a 5-mm internal diameter trephine drill (S.I.N. Implant System, São Paulo, SP, Brazil). Anesthetic and surgical procedures were followed according to the protocol reported above, and all sheep were subsequently returned to the farm, where they completely recovered after the biopsies.

Immediately after the collection, the samples containing bone and implants were fixed in 4% neutral-buffered formalin solution for 48 h. The dehydration of samples in ascending alcohol solutions of 60%, 70%, 90%, and 100% was performed under agitation and was subsequently infiltrated through daily changes of ascending grades of alcohol/resin (Technovit 7200 VLC; Kultzer, Wehrheim, Hesse, Germany): 70/30, 50/50, 30/70, and 100% resin. Thereafter, embedding of specimens into resin (Technovit 7200; Kulzer, Wehrheim, Hesse, Germany) was performed using a light polymerization unit (EXAKT 520; Exakt System, Norderstedt, Hamburg, Germany) in 2 steps by different wavelengths (white/blue light) 8 h for each light. The bone blocks were cut in the mid-axial and apical-coronal planes using a macro-scale cutting and grinding technique (Exakt 310 CP series; Exakt System, Norderstedt, Hamburg, Germany). The obtained slices were ground and polished to a final thickness of 30 to 40 µm^38^. Finally, the slices were stained with toluidine blue to differentiate newly formed bone, and acid fuchsin was used to contrast the background. Light microscopy at 10 × and 20 × magnifications (Olympus BX43; Olympus Corporation, Tokyo, Japan) supported the analysis of the slices, with images acquired using Olympus Cellsens (cellSens software; Olympus Corporation, Tokyo, Japan).

The histomorphometric analysis was conducted from reconstructions of the implant and adjacent bone. These images were obtained from captured photomicrographs with 10 × magnification in sequenced fields to scan and reconstruct. After the reconstruction of all images, the area of interest for BAFO evaluation was determined and drawn, from the first thread of the implant to the fourth thread’s beginning. This line delimitation was used to determine the BIC value, which was later transformed into a percentage. The implant-profile design was then duplicated and aligned at 270 µm in the long axis of the implant, thus completing the total area of interest. Image J software (National Institutes of Health, Bethesda, MD, USA) manually determined the bone area fraction occupancy (BAFO), which was later transformed into a percentage^36^. Using the reconstructed images, the presence of osteoid, woven bone, connective tissue, and vessels around the implants was also quantified. For this analysis, toluidine blue staining was used, as it allows for the color identification of osteoid and mineralized bone. The area of interest was drawn with a 200 µm box around the implant. For vessel quantification, scores from 0 to 4 from ISO 10993-6/2016 were used, where 0: no neovascularization; 1: minimal capillary proliferation focal 1–3 buds; 2: groups of 4–7 capillaries with supporting fibroblastic structures; 3: broad band of capillaries with supporting structures; and 4: extensive band of capillaries with supporting fibroblastic structures.

One single and experienced observer conducted the histologic and histomorphometric evaluations. All samples were coded, and the examiner evaluated the slides blindly with respect to the experimental group and endpoints.

### Statistical analysis

A Shapiro–Wilk test was used to check data distribution. The log transformation of ISQ was used to conform to normality. Fitting a normal distribution, the groups and the healing time points were compared using a t-test considering a significance level of 0.05. All analyses were accomplished using Prism Graph Pad 8.3 software (GraphPad Software, San Diego, CA, USA). The values for IT are presented in minimum, median, and maximum, and the data for BIC and BAFO are presented in mean plus confidence interval at 95% of significance.

## Data Availability

The datasets generated during and/or analyzed during the current study are available from the corresponding author on reasonable request.
